# Emerging insights into enhancer RNAs: biogenesis, function, mechanism, and disease implication

**DOI:** 10.1093/bib/bbaf700

**Published:** 2026-02-07

**Authors:** Meiqian Qiu, Xiuchong Yu, Heting Liu, Yuezhu Tao, Chunhui Huang, Yang Xi, Qi Liao

**Affiliations:** School of Public Health, Health Science Center, Ningbo University, 818 Fenghua Road, Jiangbei District, Ningbo, Zhejiang Province 315211, China; Department of Gastroenterology, The First Affiliated Hospital of Ningbo University, 59 Liuting Street, Haishu District, Ningbo, Zhejiang Province 315020, China; Department of Biochemistry and Molecular Biology, School of Basic Medical Sciences, Health Science Center, Ningbo University, 818 Fenghua Road, Jiangbei District, Ningbo, Zhejiang Province 315211, China; School of Public Health, Health Science Center, Ningbo University, 818 Fenghua Road, Jiangbei District, Ningbo, Zhejiang Province 315211, China; School of Public Health, Health Science Center, Ningbo University, 818 Fenghua Road, Jiangbei District, Ningbo, Zhejiang Province 315211, China; School of Public Health, Health Science Center, Ningbo University, 818 Fenghua Road, Jiangbei District, Ningbo, Zhejiang Province 315211, China; Department of Biochemistry and Molecular Biology, School of Basic Medical Sciences, Health Science Center, Ningbo University, 818 Fenghua Road, Jiangbei District, Ningbo, Zhejiang Province 315211, China; School of Public Health, Health Science Center, Ningbo University, 818 Fenghua Road, Jiangbei District, Ningbo, Zhejiang Province 315211, China

**Keywords:** enhancer, eRNAs, biogenesis, function, mechanism, detection, resources, disease implication

## Abstract

In current years, the molecular mechanisms through which enhancer-derived noncoding RNAs—enhancer RNAs (eRNAs) have been increasingly elucidated. eRNAs are not mere as transcript products but considered as a marker of active enhancers, usually characterized by bidirectional transcription, lack of poly(A) tails and rapid degradation. Multiple lines of evidence have demonstrated eRNAs are involved in a number of regulation processes such as enhancer-promoter (E-P) looping, recruitment of transcriptional co-activators, regulation of RNA polymerase II (Pol II) pause release, and induction of R-loops, thereby enabling precise control of gene expression. Besides, the tissue-specific regulatory mechanisms of eRNAs in diverse diseases such as cancer, cardiovascular diseases, and neurodegenerative disorders are being progressively elucidated, offering potential novel targets for precision diagnosis and therapy. This review systematically synthesizes recent advances in eRNA research, elucidating structural features, transcription processes, functions, regulatory mechanisms, detection, data resources, disease associations and proposing promising prospects for eRNA-targeted precision therapeutics.

## Introduction

The human genome comprises ~3 billion DNA base pairs, with only ~2% of the sequence directly encoding proteins [1, 2]. For a long time, the noncoding regions that account for 98% of the human genome were considered “junk DNA” because they appeared to lack protein-coding functions [3, 4]. However, emerging evidence has demonstrated that noncoding regions harbor a diverse array of crucial functional elements that play indispensable roles in gene regulation and chromatin architecture. Among these, enhancers represent a pivotal class of noncoding regulatory elements. They exert decisive influence on critical biological processes such as cellular differentiation, development, and environmental response, primarily by recruiting transcription factors and co-factors to distally regulate the expression levels of downstream target genes.

As research deepens, enhancers have been found to be transcribed, generating a class of noncoding RNAs termed enhancer RNAs (eRNAs). eRNA expression usually exhibits lower abundance and a shorter half-life than other ncRNAs. However, according to studies in recent years, eRNAs are no longer viewed merely as transcriptional byproducts. Instead, they have been substantiated to play active and indispensable roles within enhancer-mediated regulatory networks governing target genes. These roles encompass participation in chromatin conformational remodeling, assembly and stabilization of the transcription complexes, and the recruitment or modulation of transcription factor activity. Consequently, eRNA expression has become widely recognized as one of the most reliable molecular signatures of active enhancers.

Due to the important role of eRNAs in gene regulation, eRNAs have been found to participate in the pathogenesis of various human diseases such as cancer, neurodegenerative disorders, and autoimmune diseases. Given the significance and frontier nature of eRNA research, this review aims to systematically synthesize current advances in the eRNA field. We will focus on elucidating the biogenesis (formation), diverse functions, molecular mechanisms, identification strategies and technologies, database sources and the roles and potential clinical relevance of eRNAs in human diseases.

## Structural features and transcriptional processes of enhancers

### Basic characteristics of enhancers

An enhancer is a DNA sequence that enhances transcriptional activity by interacting with the promoter to increase the frequency and efficiency of transcription. The genomic localization of enhancers relative to their target genes is highly variable: upstream, downstream, or within intronic regions [5]. They not only regulate proximal genes but also modulate transcription at distal genomic loci. Furthermore, individual enhancers can coordinate the expression of multiple genes, underscoring the complexity of gene regulatory networks [6].

Compared to other regulatory elements, enhancers exhibit the following defining characteristics: (i) Sequence-Level Features: Enhancers contain abundant transcription factor (TF) binding sites with specific motifs, which contain many evolutionarily conserved sequences [7, 8]. (ii) Epigenetic Signatures: Enhancers display low DNA methylation levels and distinct histone modification patterns [9]. For example, poised enhancers are marked by co-occurring modifications H3K4me1 (monomethylation of histone H3 lysine 4) and H3K27me3 (trimethylation of histone H3 lysine 27) [7, 10]. Among them, H3K4me1 is a hallmark of enhancers, facilitating enhancer-promoter (E-P) interactions during cellular differentiation, while H3K27me3 is primarily linked to transcriptional repression [11, 12]. In contrast, active enhancers are further defined by the combinatorial presence of H3K4me1 and H3K27ac (acetylation of histone H3 lysine 27), in which H3K27ac can enhance chromatin accessibility [13]. (iii) Transcriptional Activity: A hallmark of active enhancers is bidirectional transcription, generating eRNAs as functional noncoding regulatory transcripts [14]. (iv) Chromatin Loop Architecture: Active enhancers drive gene transcription by forming chromatin loops through long-range interactions with the promoters of their target genes.

Enhancers can be classified into typical enhancers (TEs) and super enhancers (SEs) based on their functional strength, structural features, and regulatory scope. SEs, also termed enhancer clusters, are large genomic domains composed of densely packed enhancer units that critically regulate gene expression and cellular functions. Unlike TEs, SEs are characterized by pronounced enrichment of the Mediator complex subunit MED1 and high levels of the histone modification H3K27ac [15, 16]. These attributes confer SEs on enhanced regulatory capacity and broader genomic influence.

### Transcriptional processes of eRNAs

In eukaryotes, double-helical DNA is wrapped around histone octamers to form nucleosomes, which are the fundamental structural units of chromatin. This hierarchical compaction drastically reduces the physical length of DNA, enables its packaging within the confined nuclear space, and thus ensures genomic stability [17]. Simultaneously, it also imposes spatial constraints on gene regulation. However, active enhancers circumvent these spatial limitations: their transcriptional activation often precedes the synthesis of downstream target genes and eRNA transcription is thus often used as a dynamic biomarker of enhancer activity [18, 19]. The process of eRNA transcription can be delineated into the following five sequential stages.

**(1) Histone modifications.**

**Figure 1:**
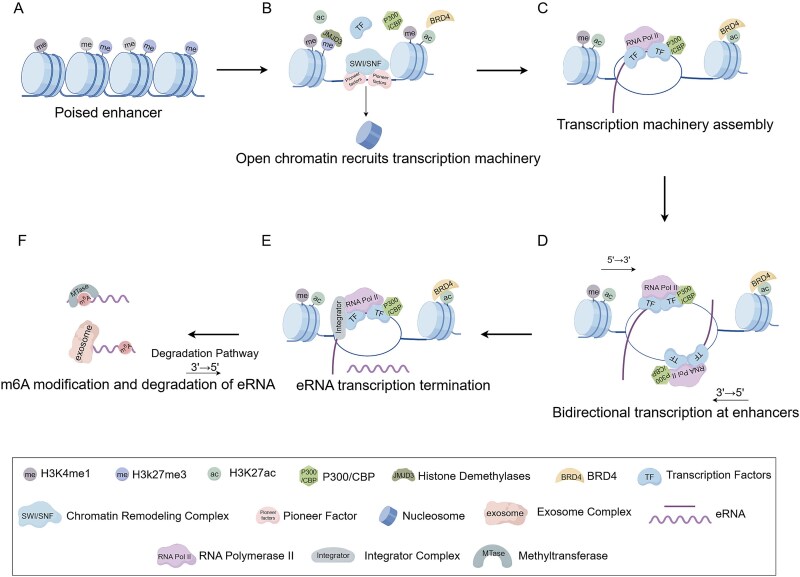
Mechanisms of enhancer transcription. (A) A poised enhancer in an inactive state.(B) Pioneer factors and chromatin remodeling complexes facilitate chromatin opening. (C) Recruitment of the transcriptional machinery following DNA unwinding at the enhancer. (D) Bidirectional transcription of the enhancer, generating sense and antisense eRNA strands. (E) Transcription termination mediated by the integrator complex. (F) eRNAs degradation via the RNA exosome complex and deposition of m6A modifications.

Histone modifications exhibit high dynamism and diversity, serving as a core mechanism of gene expression regulation. The production of eRNAs is widely recognized as a hallmark event of active enhancers. Canonical active enhancers are enriched with H3K4me1 and H3K27ac modifications, whereas poised enhancers frequently harbor the repressive H3K27me3 mark (Fig. 1A). Although both H3K27ac and H3K27me3 target the lysine 27 residue of histone H3 (H3K27), they establish fundamentally opposing chromatin states: H3K27ac promotes an open chromatin conformation, while H3K27me3 maintains a repressive, closed architecture. During enhancer activation, demethylase-mediated clearance of H3K27me3 (e.g. by JMJD3) and acetyltransferase-catalyzed deposition of H3K27ac (e.g. by p300/CBP) act synergistically to establish the epigenetic foundation required for eRNA transcription [10, 20, 21] (Fig. 1B).

**(2) Chromatin remodeling.**

Enhancer activation critically depends on dynamic chromatin accessibility. When regulatory elements compete with nucleosomes for DNA binding, the chromatin state of the regulatory elements’ core regions transitions from closed to open. This process is mainly orchestrated by pioneer factors (e.g. FOXA1, SOX2, and AP-1) and chromatin remodeling complexes (e.g. SWI/SNF). Pioneer transcription factors scan nucleosome surfaces to recognize exposed DNA motifs and enable specific binding to silent genomic regions inaccessible to other transcription factors. These factors subsequently recruit chromatin remodeling complexes, which utilize energy derived from ATP hydrolysis to drive nucleosome sliding, histone eviction, or histone variant exchange. This collaborative action ultimately opens chromatin structure and lays the foundation for transcriptional machinery assembly [22–24] (Fig. 1B). For instance, AP-1 recruits the SWI/SNF complex to nucleosome-occupied enhancers, synergistically enhancing chromatin accessibility [25, 26].

**(3) Transcriptional initiation and termination.**

Active enhancers undergo bidirectional transcription, producing both sense and antisense eRNA strands [14]. During enhancer transcription, open chromatin architecture recruits a multitude of proteins to assemble transcriptional machinery that regulates transcription [27] (Fig. 1C).

Upon DNA strand separation, TF DNA-binding domains (DBDs) anchor to open chromatin, recruiting coactivators that modify chromatin or interact with core transcriptional components. The Mediator complex, a core component of the transcription machinery, not only serves as a molecular bridge connecting TFs with RNA polymerase II (Pol II) but also directly orchestrates the recruitment of Pol II to transcriptional start sites. Through this mechanism, Pol II achieves precise initiation of transcription at specific genomic loci [4, 28, 29] (Fig. 1D).

However, Pol II cannot elongate indefinitely along DNA templates. The Integrator complex interacts with the C-terminal domain (CTD) of Pol II and plays a critical role in transcription termination (Fig. 1E). That is, Integrator cleaves nascent RNAs using its endoribonuclease activity to trigger Pol II termination and release [30, 31].

**(4) Processing of nascent eRNAs.**

The functionality of eRNAs considerably depends on dynamic post-transcriptional processing. Nascent eRNAs differ markedly from lncRNAs: they are typically unspliced, lack 3′ polyadenylation, possess 5′ caps, and are short (~200 nt-2 kb) with nuclear retention [18, 31–33]. lncRNAs are typically characterized by splicing, polyadenylation, and considerable length. Recent studies have revealed that a subset of enhancer-derived RNAs (eRNAs) can be surprisingly long (up to ~5 kb) and may also undergo splicing and polyadenylation [18]. These findings have prompted researchers to classify such molecules as enhancer-associated long noncoding RNAs (lnc-eRNAs) [18, 34].

In addition to evading splicing and polyadenylation, nascent eRNA transcripts undergo diverse chemical modifications, with m^6^A ranking among the most prevalent RNA modifications [35]. Characterized by selective m^6^A deposition i.e. catalyzed by methyltransferase complexes such as METTL3/METTL14, m^6^A modification positively correlates with enhancer activity and serves as a key epigenetic marker of active enhancers [35, 36] (Fig. 1F). They further revealed that m6A-modified eRNAs can recruit the nuclear reader protein YTHDC1. By binding to the m6A mark, this protein facilitates their partitioning into droplet-like condensates, thereby promoting phase separation. This mechanism subsequently accelerates the formation of condensates by the transcriptional activator BRD4, ultimately leading to gene activation [35]. Thus, chemical modifications of eRNAs play a critical role in enhancer activation and the regulation of gene transcription.

**(5) eRNA degradation.**

eRNAs exhibit low abundance and high susceptibility to degradation within cells. The degradation of eRNAs is primarily mediated by the RNA exosome complex, which comprises a nine-subunit core, including six distinct proteins that form a ring-like structure and three RNA-binding domain proteins that form a cap domain. The cap domain proteins are crucial for stabilizing the core structure [37]. For example, a study has demonstrated that knockdown of the RNA exosome complex increases eRNA stability but significantly impairs the ability of TF YY1 to be recruited to enhancer regions [8]. This indicates that the RNA exosome complex plays a pivotal role in eRNA degradation while potentially modulating transcription factor occupancy at enhancer regions.

Notably, excessive accumulation of eRNAs will induce potential risks to the cell by driving aberrant formation of pathogenic structures such as R-loops, and thus trigger genomic instability [38]. The experiments in vitro found that depletion of the RNA exosome results in the accumulation of R-loop structures at eRNA-expressing loci [37]. Therefore, the RNA exosome not only facilitates eRNA degradation but may also maintain chromatin accessibility at enhancer regions by clearing aberrant RNA–DNA structures, thereby indirectly supporting TF binding.

## Functional properties of eRNAs

eRNAs participate in fine-tuned regulation of gene transcription by facilitating long-range chromatin interactions between enhancers and promoters. The production of eRNAs plays a critical role in gene regulation, contributing to the stability of genetic programs. In summary, the functional mechanisms of eRNAs can be categorized into the following five principal aspects.

### Participation in E-P loop formation

Enhancers and their target gene promoters are often separated by considerable linear genomic distance. eRNAs may bridge the enhancer and promoter through physical cross-linking or by binding proteins to form complexes, thereby establishing and stabilizing E-P loops. The formation of E-P loops greatly enhances the efficiency of enhancer-mediated target gene transcription and provides both the structural and material basis for the precise regulation of gene expression [32].

Recent studies suggest that the formation of such chromatin loops may depend on a complementary pairing mechanism involving repetitive elements [39]. Specifically, eRNAs transcribed from enhancers and upstream antisense RNAs (uaRNAs) generated from promoter regions are enriched in Alu repeat sequences. The reverse complementary regions can base-pair to form RNA duplex structures and the Alu-mediated RNA duplexes act as “molecular bridges,” facilitating the recognition between enhancers and promoters and the formation of chromatin loops [39].

Concurrently, to overcome spatial constraints, CTCF and the cohesin complex organize linear DNA into looped structures via the loop extrusion mechanism. This process is dynamically regulated by eRNAs. For instance, in ERα-positive breast cancer cells, estrogen-induced specific eRNAs directly bind subunits of the cohesin complex. This interaction stabilizes cohesin’s chromatin occupancy, promoting E-P loop assembly. Knockdown experiments of these eRNAs confirmed a significant reduction in chromatin-bound cohesin levels, providing direct evidence for the physical scaffolding function of eRNAs [40].

**Figure 2:**
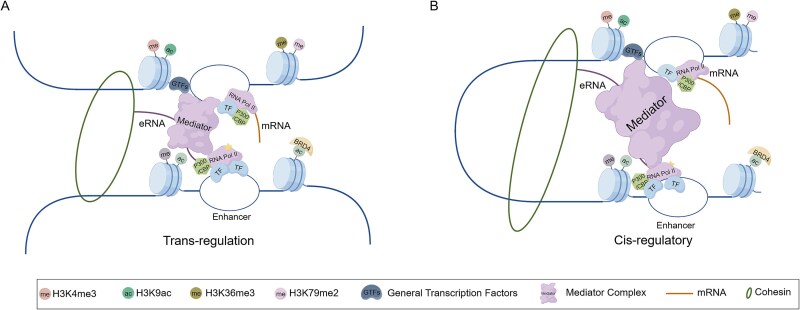
Enhancer regulatory mechanisms. (A) Enhancers mediate trans-regulatory interactions with promoters on different chromosomes. (B) Enhancers mediate cis-regulatory control of promoters on the same chromosome.

Critically, the regulatory repertoire of eRNAs extends beyond the classical cis-acting paradigm, encompassing trans-regulatory functions [41]. A compelling example is observed in mouse myoblasts, where two distinct eRNAs transcribed from an enhancer region on chromosome 7 exhibit differential regulatory scopes: ^CE^eRNA modulates transcription of the adjacent MyoD gene on the same chromosome (cis-regulation) (Fig. 2A), whereas ^DRR^eRNA regulates the Myogenin gene located on chromosome 1, demonstrating definitive trans-regulation [42] (Fig. 2B). The formation of E-P chromatin loops significantly amplifies the efficiency of enhancer-mediated transcriptional control. This three-dimensional chromatin architecture serves as a fundamental structural platform and essential physical entity, and thus enables precise spatiotemporal regulation of gene expression. Concurrently, a similar regulatory mechanism was identified in developing mouse blastocysts: the eRNA derived from the enhancer p-Enh can not only regulate the neighboring gene Cdx2 in cis but also, via a trans-regulatory mechanism, interact with the SMAD4 protein. This interaction participates in the TGF-β signaling cascade, thereby modulating the global transcriptome and epigenomic landscape [43].

### Spatial orchestration of target gene activation

**Figure 3:**
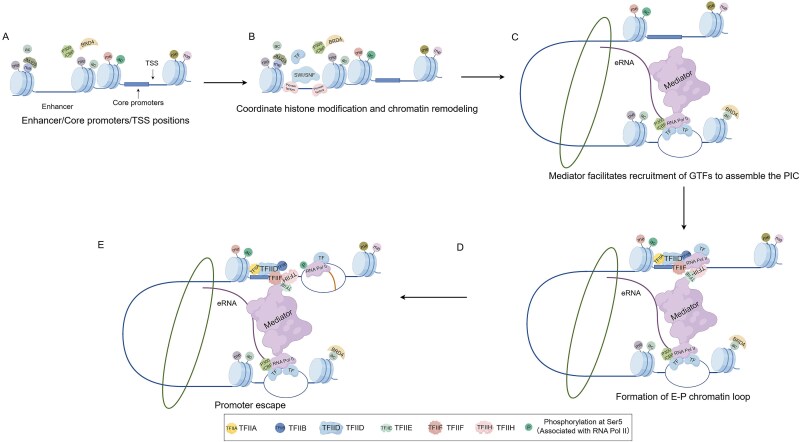
E-P loop formation process. (A) Localization of enhancer, core promoter, and transcription start site (TSS). (B) Enhancer activation. (C) Mediator complex bridges Pol II and general TFs (GTFs). (D) Preinitiation complex (PIC) assembly completed with enhancer-promoter (E-P) loop formation. (E) Promoter clearance and transition to transcriptional elongation.

During chromatin looping, eRNAs stabilize interactions between the Mediator complex and enhancers through direct binding. Functioning as a molecular bridge connecting Pol II with general transcription factors (GTFs), Mediator undergoes phase separation upon binding to activation domains (ADs) of enhancer-bound transcription factors, forming dynamic transcriptional condensates. This liquid–liquid phase-separated microenvironment significantly elevates local protein concentration within E-P regions and co-localizes Pol II, GTFs (e.g. TFIIA, TFIIB, TFIID, TFIIE, TFIIF, and TFIIH), and mediator at core promoter sites. Consequently, it cooperatively facilitates functional assembly of the pre-initiation complex (PIC), and thereby activates transcription of target genes [28, 44, 45] (Fig. 3).

### Recruitment and stabilization of transcription factors binding

**Figure 4:**
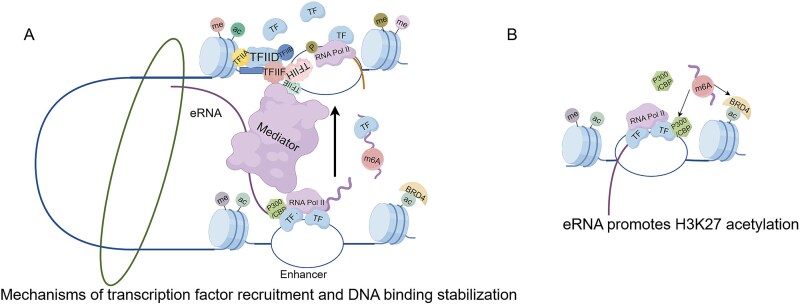
Mechanistic roles of eRNAs. (A) eRNAs interact with transcriptional machinery (TFs, Mediator, cohesin, Pol II) to stabilize TF-DNA binding. (B) eRNAs bind p300/CBP’s HAT domain and BRD4’s bromodomain to promote open chromatin.

As we known, TFs regulate gene expression by specifically recognizing DNA cis-regulatory elements. eRNAs can further amplify this process through dual regulatory mechanisms. On one hand, eRNAs directly bind TFs (e.g. YY1), forming RNA-protein complexes that guide their targeting to enhancer or promoter regions. On the other hand, eRNAs significantly enhance the binding stability of TFs by prolonging their interaction time with DNA, establishing a positive feedback regulatory loop (Fig. 4A). For instance, in mouse cells, the TF YY1 binds eRNAs. This RNA-protein interaction markedly increases TF occupancy at enhancer regions, cooperatively activating the transcriptional program of downstream target genes [8]. A similar mechanism operates in unexplained recurrent pregnancy loss (URPL). The eRNA lnc-SLC4A1–1 recruits NF-κB and stabilizes its binding to the promoter region of the pro-inflammatory gene CXCL8 (encoding IL-8). This induces the enrichment of the histone modification mark H3K27ac, thereby activating CXCL8 transcription [46].

### Enhancing chromatin accessibility

CBP and p300, highly homologous transcriptional coactivators, both possess histone acetyltransferase (HAT) domains. Their binding with enhancers catalyzes H3K27ac, thereby promoting chromatin relaxation and augmenting enhancer transcriptional activity [21].eRNAs recruit CBP/p300 and interact with these coactivators to modulate local histone modifications and facilitate an open chromatin configuration (Fig. 4B). For example, the antisense eRNA Khps1—transcribed from a downstream enhancer—recruits CBP/p300 to the SPHK1 promoter. This recruitment induces alterations in local chromatin architecture, thereby facilitating the binding of transcription factor E2F1 and activating oncogenic signaling pathways [47]. Furthermore, biochemical and cellular studies demonstrate that eRNAs binding to the HAT domain of CBP directly stimulates its acetyltransferase activity, and thus modulating H3K27ac levels [48, 49].

Additionally, BRD4 is another critical coactivator that regulates chromatin accessibility. eRNAs can also directly bind to BRD4, enhancing BRD4’s specific recognition of acetylated histones (H3K27ac). This interaction facilitates the recruitment of transcriptional complexes (e.g. Mediator-Pol II), thereby augmenting enhancer-driven transcription efficiency [50].

### Regulating Pol II pause-release

**Figure 5:**
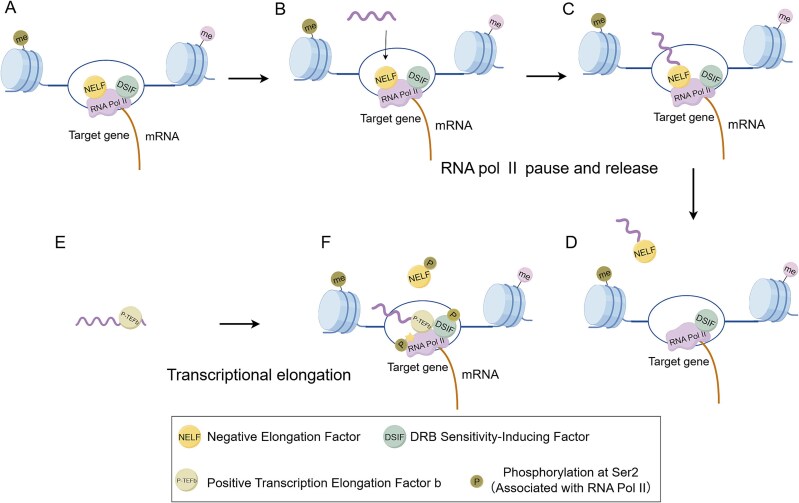
eRNAs-mediated regulation of Pol II pause-release. (A) NELF/DSIF enforce pol II pausing. (B) eRNAs competitively bind NELF.(C) eRNAs-NELF complex formation. (D) Complex dissociation enables elongation. (E) eRNAs activates P-TEFb. (F) eRNAs-NELF competition facilitates Pol II phosphorylation (Ser2p) and elongation.

During eukaryotic transcription, the elongation phase of Pol II is critical for the efficient transfer of genetic information from DNA to RNA. Shortly after transcription initiation, Pol II is subjected to opposing regulatory inputs from the negative elongation factor complex (NELF) and the positive transcription elongation factor b complex (P-TEFb) [51]. NELF (a heterotetramer composed of NELFA, NELFB, NELF C/D, and NELFE) and DSIF (SUPT5H/SUPT4H) associate with Pol II to form the Pol II-DSIF-NELF complex. This complex induces transcriptional pausing, impeding the production of nascent RNA. Conversely, P-TEFb (comprising cyclin-dependent kinase 9 (CDK9) and cyclin T1 (CCNT1) phosphorylates the Ser2 residue within the carboxy-terminal domain (CTD) of Pol II (Pol II-Ser2p), which indirectly triggers NELF dissociation, thereby promoting the transition of paused Pol II into productive elongation [52–55] (Fig. 5).

Mounting experimental evidence underscores the central regulatory functions of eRNAs in modulating Pol II pause-release dynamics. Gorbovytska V et al. demonstrated that eRNAs exceeding 200 nucleotides in length and containing unpaired guanine bases can form multivalent and heterogeneous contacts with the NELF-E and NELF-A subunits, which effectively trigger NELF release, thereby facilitating the elongation of paused Pol II [56]. For instance, a study in neuronal cells demonstrates that eRNAs function as molecular decoys for NELF, thereby promoting the transition of paused Pol II into productive elongation [56]. Moreover, in a study of castration-resistant prostate cancer (CRPC), PSA eRNA activates P-TEFb by binding to the CYCLIN T1 subunit of the P-TEFb complex, significantly enhancing Pol II-Ser2 phosphorylation (Ser2p) and thereby promoting transcriptional elongation [57].

### R-Loop formation

R-loops are three-stranded nucleic acid structures composed of an RNA:DNA hybrid and a displaced single-stranded DNA molecule. They are prevalent throughout the cell cycle, and the dynamic equilibrium between their formation and resolution is critical for genomic stability [58, 59].

Based on their biological consequences, R-loops can be categorized as either “physiological” or “pathological”. Physiological R-loops play essential roles in DNA repair, replication, and transcription, contributing to cellular homeostasis and normal function. Conversely, dysregulation of cellular control mechanisms or erroneous formation of R-loops can lead to pathological R-loops. These pathological structures compromise genomic stability and can impede DNA replication, transcription, and repair processes [41, 59–61].

Recent studies have revealed a close association between eRNAs and R-loops, highlighting their significant role in gene expression regulation [32, 62]. For example, researchers from the University of Southern California discovered that a long noncoding enhancer RNA (lnc-eRNA) derived from the Npas4 locus forms an R-loop structure. This R-loop supports the formation of a three-dimensional chromatin loop between the enhancer and its cognate promoter, thereby facilitating rapid induction of Npas4 gene expression. Notably, this specific lnc-eRNA and its associated R-loop are essential for the adaptive behavioral changes induced by chronic psychosocial stress or cocaine exposure [34]. Similarly, in the regulation of the Pcdhα gene cluster, the antisense eRNA PEARL transcribed from the HS5–1 enhancer forms local R-loop structures. By inhibiting the CTCF/cohesin-mediated “loop extrusion” process, it anchors the distal enhancer to specific variable promoters (e.g. Pcdhα6/α12), establishing stable three-dimensional chromatin interactions. This mechanism thereby activates stochastic expression of the neural identity code gene Pcdhα, ultimately determining single-neuron connection specificity in the brain [63].

## Identification of eRNAs

### Genome-wide identification of eRNAs

Advances in high-throughput sequencing technologies have significantly facilitated the detection of eRNAs. As direct transcriptional products of active enhancers, eRNAs require integrated analysis of both the characteristics of their genomic-derived enhancers and the properties of themselves for precise identification. Active enhancers typically coincide with an open chromatin state, which can be mapped using ATAC-seq. Moreover, key hallmark features of active enhancers, such as the histone modification H3K27ac or binding of the co-activators CBP/p300, can be comprehensively analyzed via ChIP-seq. Critically, leveraging the established mechanism whereby enhancers regulate target gene expression through chromatin looping, chromatin conformation capture technologies (e.g. 4C-seq, 5C-seq, Hi-C, and Capture Hi-C) can be applied to map candidate active enhancer regions engaging in spatial interactions with target gene promoters (Table 1).

**Table 1:** Summary of eRNA identification methods.

**Fundamental characteristics**	**Name**	**Principle**	**Advantages**	**Limitations**	**References**
Genome-wide identification of eRNAs	ATAC-seq	Tn5 transposase inserts sequencing adapters into open chromatin regions, followed by high-throughput sequencing.	(1) Low input requirements (2) Rapid workflow	(1) Cannot definitively distinguish enhancers from promoters based on accessibility alone	[4, 64, 65]
	ChIP-Seq	Antibody-based enrichment of protein-bound DNA fragments for sequencing.	(1) High spatial resolution (2) Low background noise (3) Genome-wide coverage	(1) Dependent on antibody specificity	[66, 67]
	4C	3C variant using inverse PCR + NGS to profile interactions from a single locus.	(1) Detects long-range contacts (2) High sensitivity	(1) Difficulty distinguishing biological interactions from PCR artifacts	[68]
	5C	Multiplexed ligation-mediated amplification of 3C libraries.	(1) High resolution (2) Effective fordistal element detection	(1) Probe hybridization efficiency exhibits variability. (2) The accurate assessment of sensitivity poses a challenge.	[68, 69]
	Hi-C	Genome-wide chromatin architecture mapping via biotin-labeled ligation and deep sequencing.	(1) Perform in situ proximity ligation within intact nuclei. (2) Generate a genome-wide interaction map.	(1) Limited spatial resolution (2) Lacks temporal resolution	[70, 71]
	Capture Hi-C	Probe-based enrichment of specific regions post Hi-C library construction.	(1) Enables targeted, high-resolution analysis of chromatin interactions in specific genomic regions	(1) Probe design must consider both sensitivity and specificity. (2) Lacks genome-wide context	[72]
	ChIA-PET	Combines ChIP with proximity ligation to map protein-anchored interactions.	(1) Links interactions to specific DNA-binding proteins	(1) Low sensitivity for low-abundance proteins (2) Primarily detects cis-interactions	[73–75]
Transcriptome-wide identification of eRNAs	RamDA-seq	Unbiased total RNA capture coupled with full-length cDNA synthesis enables highly sensitive profiling of diverse non-polyadenylated short non-coding RNAs, including eRNAs.	(1) Comprehensive RNA coverage (2) High sensitivity	(1) Complex experimental protocol. (2) The data exhibited a high rRNA content.	[76]
	GRO-seq/PRO-seq	Nascent RNA sequencing to map RNA polymerase II occupancy in real-time.	(1) Captures ongoing transcription (2) Base-pair resolution.	(1) Technically demanding (2) Requires large numbers of input cells.	[77, 78]
	BruUV-seq	BrdU labeling + UV crosslinking for nascent RNA pulldown and sequencing.	(1) High sensitivity and low background noise (2) Direct detection of nascent transcription	(1) High complexity and cost (2) Potential cytotoxicity (3) Technical biases	[78]
Experimental Approaches for eRNA Detection	RNA FISH	Fluorescent probe hybridization for eRNA visualization in fixed cells/tissues.	(1) Subcellular spatial resolution (2) High sensitivity (3) Multiplexing capability	(1) Low throughput (2) Semi-quantitative	[79]
	Nuclear-Cytoplasmic Fractionation	Physical separation of cellular compartments to analyze eRNA localization.	(1) Simple workflow (2) Subcellular distribution data	(1) Low sensitivity (2) Risk of eRNA degradation (3) Cross-contamination potential	[80]
	RIP	Antibody-mediated enrichment of endogenous eRNA-protein complexes to isolate target RNA interactomes.	(1) High physiological relevance (2) Target-specific association	(1) High background binding risk (2) Low throughput	[81]
	Perturbation Experiments	Functional disruption of eRNAs(e.g. CRISPRi, ASOs) to assess transcriptional and phenotypic impacts.	(1) Establishes functional causality (2) High target specificity	(1) Confounded by off-target effects (2) Variable delivery efficiency	[82]

### Transcriptome-wide identification of eRNAs

At the transcript level, the distinctive properties of eRNAs provide critical dimensions for their identification: Their characteristic lack of poly(A) tails enables screening and identification of potential eRNAs transcripts while avoiding poly(A) enrichment—from total RNA-seq data [32]. As unstable nascent eRNAs frequently exhibiting bidirectional transcription patterns, eRNAs can be resolved with high resolution through GRO-seq or PRO-seq methodologies that precisely capture RNA polymerase II position, density, and transcriptional directionality. Furthermore, the BruUV-seq technique achieves specific and sensitive capture of these labile nascent eRNAs by integrating BrdU metabolic labeling, UV-induced damage, and immunoaffinity enrichment strategies. Consequently, reliable eRNA identification cannot rely on single-dimensional approaches but necessitates integrated application and multidimensional analysis of the aforementioned methodologies—combining both enhancer-associated features (e.g. chromatin accessibility, histone marks) and transcript-specific characteristics (e.g. non-polyadenylation, nascent transcription dynamics). Only through such combinatorial frameworks can precisely screening and definitive validation of active enhancer-derived eRNAs be accomplished (Table 1).

### Experimental approaches for eRNA detection

eRNAs are predominantly localized within the nuclear compartment [83]. Their transcriptional loci can be visually mapped using RNA fluorescence *in situ* hybridization (RNA FISH). Furthermore, combining nuclear-cytoplasmic fractionation with downstream RNA detection methods (e.g. RT-qPCR) definitively validates nuclear enrichment of eRNAs. Given that eRNAs frequently associate with transcription factors and other proteins, RIP assays can be employed to co-capture eRNAs and their interacting protein partners. Based on eRNA functional properties, perturbation experiments—such as direct eRNA knockdown using ASOs/siRNAs or modulation of their source enhancer activity via CRISPRi/CRISPRa—enable investigation of regulatory functions. By combining these perturbations with RT-qPCR-based quantification of both eRNA expression levels and those of their putative target genes, the regulatory roles of eRNAs can be comprehensively elucidated (Table 1).

## eRNAs data resources

The rapid advancement of genomic technologies and the establishment of multidimensional functional annotation systems have significantly deepened our understanding of genomic regulatory mechanisms and the molecular basis of disease.

In recent years, a series of databases focused on human eRNAs have been established: in the aspect of foundational annotation, HeRA and TCeA systematically construct genomic coordinate maps and cross-tissue expression profiles of enhancers/super-enhancers through integration of resources including GTEx and ENCODE. As for regulatory mechanism analysis, eRNAbase consolidates data from ENCODE, GEO/SRA, and similar sources, providing not only comprehensive epigenetic and genetic annotations for eRNAs but also supporting eRNA-mediated pathway regulation analysis, variant interpretation annotation, and transcription factor-target gene regulatory analysis. eRNAs-IDO implements end-to-end analytical workflows—encompassing eRNA identification, interactome mining, and functional annotation—via integrated analysis of cross-scale data from GTEx, TCGA, and FANTOM5, thereby providing comprehensive support for pathophysiological investigations. In precision medicine applications, the eRic database innovatively links CTRP clinical trial records with GDSC drug sensitivity resources to develop eRNA-drug response prediction algorithms for guiding targeted therapies. The GPIeR platform establishes clinical prognostic models between eRNA expression and tumor immune phenotypes by leveraging TCGA multi-omics data and the immune microenvironment analysis tool TIMER. The eaQTLdb leverages TCGA multi-omics data to facilitate the prioritization of candidate genetic variants and enhancers, meanwhile elucidating the roles of eaQTLs in dysregulated cancer pathways and the tumor immune microenvironment. The CancereRNAQTL database amalgamates data from TCGA and other sources to investigate the effects of single nucleotide polymorphisms (SNPs) on tumor eRNA expression. Collectively, these databases constitute an evolving toolkit that bridges fundamental characterization to clinical translation in eRNA research (Table 2).

**Table 2:** Summary of human eRNAs databases.

**Database**	**Year**	**URL**	**Purpose**	**Key features**	**References**
**eRic**	2019	https://hanlab.uth.edu/eRic	Profile eRNAs expression, function, and drug responses across cancer types	(1) Quantify cancer-specific eRNAs. (2) Predict TF binding and pathway impacts. (3) Link eRNAs to clinically actionable genes (CAGs) and immune checkpoints (IC s). (4) Predict functional roles in tumorigenesis. (5) Identify clinically relevant eRNAs. (6) Explore cancer-associated eRNAs networks.	[84]
**HeRA**	2020	https://hanlab.uth.edu/HeRA/	Characterize eRNAs expression landscapes, regulatory networks, and trait associations across human tissues	(1) Quantify eRNAs expression. (2) Identify trait-associated eRNAs. (3) Predict eRNAs-associated TFs. (4) Predict eRNAs-target genes.	[85]
**TCeA**	2020	https://bioinformatics.mdanderson.org/public-software/tcea	Provide high-resolution eRNAs maps to quantify super-enhancer activity	(1) Annotate super-enhancer regions. (2) Identify immunotherapy-related super-enhancer eRNAs. (3) Link eRNAs to clinical outcomes, copy number alterations, and methylation. (4) Analyze 3D eRNAs-promoter interactions.	[86]
**GPIeR**	2022	https://hanlab.tamhsc.edu/GPIeR/	Investigate eRNAs-associated genetic variants, drug responses, and immune infiltration in cancers	(1) Identify genetic variants associated with eRNAs. (2) Link eRNAs to patient survival. (3) Catalog eRNAs correlated with drug sensitivity. (4) Catalog eRNAs associated with immune cell abundance.	[87]
**eRNAbase**	2023	http://bio.liclab.net/eRNAbase/index.php	Explore multi-omics regulatory mechanisms of human/mouse eRNAs	(1) Provide detailed (epi)genetic annotations. (2) Offer three regulatory analyses: (3) eRNAs-mediated pathway regulation. (4) eRNAs-based variant interpretation. (5) eRNAs-TF-target gene networks.	[88]
**eaQTLdb**	2023	http://www.bioailab.com:3838/eaQTLdb	Explore the effects of genetic variants on enhancer activity and prioritize candidate variants across diverse cancer types	(1) Systematic identification of genetic variants modulating enhancer activity. (2) Association analysis between cancer hallmarks and patient survival outcomes. (3) Deciphering regulatory mechanisms in the tumor immune microenvironment	[89]
**eRNAs-IDO**	2024	http://bioinfo.szbl.ac.cn/eRNA_IDO/	Explore the identification, interactome discovery, and functional annotation of human eRNAs	(1) Identification and characterization of eRNAs with customizable enhancer region definition. (2) Provision of tissue−/cell-type-specific functional annotation. (3) Elucidation of eRNAs biogenesis mechanisms and functional dynamics.	[90]
**CancereRNAQTL**	2024	http://canernaqtl.whu.edu.cn/#/	Elucidation of the significance of eRNAQTLs in transcriptional regulation and disease heritability, highlighting the potential of eRNA-based therapeutic strategies for cancers	(1) Construct the panoramic landscape of eRNAQTL systems across 30 cancer types (2) Associate eRNAQTLs with patient prognosis, hallmarks of cancer, and the immune microenvironment (3) Analyze eRNAQTLs in Chinese colorectal cancer patients to establish the first Chinese eRNAQTL atlas.	[91]

## The relationship between eRNAs and disease

Beyond fundamental transcriptional regulatory mechanisms, increasing attention is being directed toward the tissue-specific involvement of eRNAs in the pathogenesis and progression of diseases. Herein, we systematically elucidate the distinct mechanistic roles of eRNAs in specific contexts such as inflammation-related and immune-related disorders, neoplastic diseases, cardiovascular pathologies, and neuropsychiatric conditions, alongside their clinical translational potential.

### Role of eRNAs in inflammatory and immune diseases

eRNAs play critical regulatory roles in inflammatory and immune disorders. In systemic inflammatory diseases, immune microenvironment dysregulation triggered by chronic infection represents a core pathological feature. For instance, *Helicobacter pylori* infection activates the NF-κB pathway, inducing synthesis of eRNAs associated with inflammatory genes (e.g. IL1A, IL1B), thereby promoting their mRNA expression and exacerbating inflammatory responses [92]. A similar pathogenic mechanism operates in inflammatory bowel disease (IBD)—a group of chronic idiopathic disorders primarily comprising Crohn’s disease (CD) and ulcerative colitis (UC) [93]. Studies demonstrate that LPS stimulation in monocytes upregulates eRNAs linked to chemokine genes (e.g. eRNAs corresponding to CXCL1–3, CXCL8). Notably, this upregulation precedes activation of the inflammatory genes themselves and significantly influences intestinal mucosal immune responses [31, 94].

Dysfunction of eRNAs is also closely associated with autoimmune diseases. In systemic lupus erythematosus (SLE), expression of the eRNAs SLEAR was reduced in peripheral blood mononuclear cells (PBMCs) due to the specific nucleotide mutations within its corresponding enhancer region. These mutations directly impair the binding capacity of the transcription factor STAT1, preventing it from effectively activating the transcription of downstream anti-apoptotic genes, such as BCL2L1. This eRNAs deficiency-induced disruption of gene regulation ultimately leads to excessive apoptosis of immune cells, which propagates the autoimmune response [95].

### Role of eRNAs in cancer

Advances in bioinformatics have revealed widespread dysregulation of eRNA expression across diverse cancer types. The pathogenesis of mixed lineage leukemia (MLL), a hematological malignancy, serves as a quintessential example. The MLL fusion protein, resulting from chromosomal translocations, significantly reprograms the enhancer landscape by forming complexes with BRD4. This subsequently leads to the aberrantly high expression of the enhancer-associated long noncoding RNA (lnc-eRNA) SEELA. SEELA directly binds to the histone H4K31 site, thereby activating the transcription of the adjacent oncogene SERINC2 in cis. This hijacking mechanism of the E-P loop underscores the pivotal role of the coordinated dysregulation involving chromatin modifiers and three-dimensional genome architecture in leukemogenesis [96].

eRNA-mediated gene regulatory mechanism is also critically important in solid tumors. For instance, In normal prostate tissue, specific eRNA, such as lactotransferrin-eRNA (LTFe), facilitates the recruitment of RNA-binding proteins (e.g. HNRNPF). This complex promotes the formation of E-P chromatin looping, thereby activating transcription of downstream tumor-suppressive target genes such as LTF (encoding lactotransferrin). The LTF protein exerts critical tumor-suppressive functions by modulating iron ion transport to promote ferroptosis. However, in prostate cancer, androgen receptor (AR) acts as a repressive transcription factor, suppresses LTFe function and downregulates expression of its target gene LTF, ultimately leading to resistance to ferroptosis and driving tumor progression [80, 97].

### Role of eRNAs in cardiovascular disease

eRNA expression and function are critically implicated in the pathogenesis and progression of cardiovascular diseases, contributing to pathological processes through modulation of key genes. In atherosclerosis (ASCVD), super-enhancer-derived ABCA1-seRNA was found to exert dual regulatory functions. In cis-regulation, ABCA1-seRNA activates transcription of its host gene ABCA1 by recruiting the transcriptional mediator MED23 and transcription factor RXRα/LXRα, promoting cholesterol efflux and high-density lipoprotein (HDL) biogenesis to reduce lipid accumulation. However, in trans-regulation, ABCA1-seRNA binds the NF-κB subunit P65 (RelA), mediating its K48-linked ubiquitination and subsequent proteasomal degradation. This P65 degradation inhibits the NF-κB pathway, suppressing M1 macrophage polarization, inflammatory cytokine release, and vascular endothelial adhesion, ultimately attenuating atherosclerotic plaque formation [98, 99].

In heart failure (HF), the expression of cardiac-specific super-enhancer-associated eRNA, such as LINC00881, is frequently downregulated and loss-of-function due to hypermethylation. LINC00881 typically acts as upstream regulators, modulating critical genes (such as calcium channel genes RYR2 and CACNA1C, and sarcomere gene MYH6) through mechanisms involving chromatin remodeling (e.g. interaction with chromatin remodelers like SMARCA4) or direct recruitment of transcription factors. The absence of its expression impairs cardiomyocyte calcium cycling, sarcomere organization, and energy metabolism pathways, thereby exacerbating cardiac functional deterioration [100].

### Role of eRNAs in neurodegenerative diseases

Dysregulated eRNAs are frequently detectable in neurodegenerative contexts. In Huntington’s disease (HD), enhancer-derived eRNAs in the striatum exhibit widespread dysregulation, which is directly linked to the loss of pol II binding at critical enhancer regions. This eRNA downregulation serves as a key mechanism underlying transcriptional dysregulation of striatal neuronal identity genes by impairing super-enhancer-mediated recruitment of pol II [101].

Alzheimer’s disease (AD) risk-associated variants within the APOE enhancer affect the function of its cognate eRNA, AANCR. The AANCR eRNA transcript (rather than the mere act of its transcription) directly promotes APOE expression by localizing near the APOE gene locus. Elevated APOE expression subsequently induces the transformation of astrocytes into a pro-inflammatory A1 state. Chronic neuroinflammation represents a core pathological hallmark of neurodegenerative diseases. Notably, under stress conditions (such as mitochondrial dysfunction or Amyloid-β aggregation), activation of the ATM-ERK-AP1 signaling pathway further drives AANCR transcription. This establishes a deleterious positive feedback loop: “Stress - Elevated AANCR expression-Elevated APOE expression-A1 astrocytes Neuroinflammation.” This loop exacerbates neuroinflammation and mitochondrial damage during disease progression [102].

## Summary and future perspectives

In this review, we summarized the structure features, transcription process, functions, regulatory mechanisms, identification, data resources and disease association of eRNA. As accumulated evidences shown, the transcription of eRNAs not only serves as a hallmark of enhancer activity but also amplifies gene expression through multifaceted mechanisms, including stabilization of TF binding, facilitation of chromatin accessibility, and modulation of Pol II pausing-release dynamics. These discoveries challenge the conventional view of enhancers as passive protein-binding platforms, revealing their complexity and versatility in transcriptional regulation.

Despite their low abundance and rapid degradation, methodologies for detecting eRNAs are continuously evolving. Multi-omics combinatorial strategies (e.g., ATAC-seq/GRO-seq/Capture Hi-C) are widely used and have significantly improved detection accuracy, while novel techniques like BruUV-seq enable specific capture of nascent eRNAs. However, because the transcriptional start and termination sites of eRNAs are often poorly defined, their experimental capture and identification remain extremely challenging. Consequently, only a limited number of eRNAs have been functionally validated. Elucidating eRNA functions remains a significant challenge.

Dysregulation of eRNAs has been established as a critical contributor to the pathogenesis of multiple major diseases. Although studies across diverse diseases consistently support a central paradigm in which eRNA dysfunction compromises genomic stability, disrupts core signaling pathways, and ultimately underpins disease pathogenesis, the specific mechanisms by which eRNAs orchestrate target gene expression and initiate pathological cascades in most diseases remain inadequately understood. This deficit in foundational research directly constrains the clinical translation of eRNAs. Currently, eRNAs have been validated as diagnostic and prognostic biomarkers in specific contexts; however, their therapeutic targeting remains confined to preclinical exploration without substantive progression to clinical practice. Consequently, a significant gap persists between existing research and the comprehensive clinical exploitation of eRNAs, necessitating intensified mechanistic and translational investigations.In summary, research on eRNAs is currently at a pivotal stage of development. Core bottlenecks—such as difficulties in experimental validation, insufficient multi-omics data resources and integrated analysis capabilities, and lagging clinical translation—represent key challenges that also delineate clear avenues for future breakthroughs.

In summary, research on eRNAs is currently at a pivotal stage of development. Core bottlenecks—such as difficulties in experimental validation, insufficient multi-omics data resources and integrated analysis capabilities, and lagging clinical translation—represent key challenges that also delineate clear avenues for future breakthroughs.

By persistently advancing technological innovation (e.g. developing higher-sensitivity detection methods and functional perturbation tools), vigorously constructing systematic data resources, deepening investigations into functional mechanisms (moving beyond correlative studies to elucidate precise molecular interactions), and actively exploring their translational applications in disease diagnosis and therapy (e.g. developing targeted strategies), we can progressively unravel more intricate regulatory mechanisms governed by eRNAs.

Key Points

- eRNAs are functional regulators, not transcriptional noise: They actively facilitate gene expression through enhancer-promoter looping, recruitment of transcription factors/co-activators, regulation of Pol II pause-release, and chromatin remodeling.
- eRNAs employ diverse mechanisms for precise gene control: Key functions include stabilizing 3D chromatin interactions, promoting transcriptional condensate formation via phase separation, enhancing TF binding stability, and modulating R-loop dynamics.
- Detecting and validating eRNAs remains challenging: Their low abundance, rapid degradation, and lack of defined start/end points necessitate integrated multi-omics approaches (e.g. ATAC-seq, GRO-seq, Hi-C) and novel techniques like BruUV-seq.
- eRNA dysregulation is a critical driver of major diseases: Aberrant eRNA expression or function contributes significantly to the pathogenesis of cancers, inflammatory/autoimmune disorders, cardiovascular diseases, and neurodegenerative conditions.

## Data Availability

This is a review article, and no new primary data were generated in support of this work. All data discussed or analyzed in this review are based on previously published studies, which are cited in the reference list. Readers can access the original datasets through the respective publications or public repositories indicated in the cited articles.

## References

[1] Cohen D . General designs reveal distinct codes in protein-coding and non-coding human DNA. *Genes* 2022;13:1970. 36360206 10.3390/genes13111970 PMC9690640

[2] Thomas HF, Buecker C. What is an enhancer? *Bioessays* 2023;45:e2300044. 37256273 10.1002/bies.202300044 PMC11475577

[3] Mattick JS . Non-coding RNAs: The architects of eukaryotic complexity. *EMBO Rep* 2001;2:986–91. 11713189 10.1093/embo-reports/kve230 PMC1084129

[4] Rickels R, Shilatifard A. Enhancer logic and mechanics in development and disease. *Trends Cell Biol* 2018;28:608–30. 29759817 10.1016/j.tcb.2018.04.003

[5] Pennacchio LA, Bickmore W, Dean A. et al. Enhancers: Five essential questions. *Nat Rev Genet* 2013;14:288–95. 23503198 10.1038/nrg3458 PMC4445073

[6] Mohrs M, Blankespoor CM, Wang ZE. et al. Deletion of a coordinate regulator of type 2 cytokine expression in mice. *Nat Immunol* 2001;2:842–7. 11526400 10.1038/ni0901-842

[7] Heintzman ND, Hon GC, Hawkins RD. et al. Histone modifications at human enhancers reflect global cell-type-specific gene expression. *Nature* 2009;459:108–12. 19295514 10.1038/nature07829 PMC2910248

[8] Sigova AA, Abraham BJ, Ji X. et al. Transcription factor trapping by RNA in gene regulatory elements. *Science* 2015;350:978–81. 26516199 10.1126/science.aad3346 PMC4720525

[9] Stadler MB, Murr R, Burger L. et al. DNA-binding factors shape the mouse methylome at distal regulatory regions. *Nature* 2011;480:490–5. 22170606 10.1038/nature10716

[10] Rada-Iglesias A, Bajpai R, Swigut T. et al. A unique chromatin signature uncovers early developmental enhancers in humans. *Nature* 2011;470:279–83. 21160473 10.1038/nature09692 PMC4445674

[11] Chen ZY, Zhang Y. Maternal H3K27me3-dependent autosomal and X chromosome imprinting. *Nat Rev Genet* 2020;21:555–71. 32514155 10.1038/s41576-020-0245-9 PMC7717073

[12] Kubo N, Chen PB, Hu R. et al. H3K4me1 facilitates promoter-enhancer interactions and gene activation during embryonic stem cell differentiation. *Mol Cell* 2024;84:1742–52. 38513661 10.1016/j.molcel.2024.02.030 PMC11069443

[13] Yang J, Zhou F, Luo X. et al. Enhancer reprogramming: Critical roles in cancer and promising therapeutic strategies. *Cell Death Discovery* 2025;11:84. 40032852 10.1038/s41420-025-02366-3 PMC11876437

[14] Ye R, Cao CC, Xue YC. Enhancer RNA: Biogenesis, function, and regulation. *Essays Biochem* 2020;64:883–94. 33034351 10.1042/EBC20200014

[15] Whyte WA, Orlando DA, Hnisz D. et al. Master transcription factors and mediator establish super-enhancers at key cell identity genes. *Cell* 2013;153:307–19. 23582322 10.1016/j.cell.2013.03.035 PMC3653129

[16] Bell E, Curry EW, Megchelenbrink W. et al. Dynamic CpG methylation delineates subregions within super-enhancers selectively decommissioned at the exit from naive pluripotency. *Nat Commun* 2020;11:1112. 32111830 10.1038/s41467-020-14916-7 PMC7048827

[17] Sahu RK, Singh S, Tomar RS. The mechanisms of action of chromatin remodelers and implications in development and disease. *Biochem Pharmacol* 2020;180:114200. 32805211 10.1016/j.bcp.2020.114200

[18] Han ZZ, Li W. Enhancer RNA: What we know and what we can achieve. *Cell Prolif* 2022;55:e13202. 35170113 10.1111/cpr.13202 PMC9055912

[19] Khor JM, Guerrero-Santoro J, Douglas W. et al. Global patterns of enhancer activity during sea urchin embryogenesis assessed by eRNA profiling. *Genome Res* 2021;31:1680–92. 34330790 10.1101/gr.275684.121 PMC8415375

[20] Zhang XX, Liu L, Yuan X. et al. JMJD3 in the regulation of human diseases. *Protein Cell* 2019;10:864–82. 31701394 10.1007/s13238-019-0653-9 PMC6881266

[21] Chen Q, Yang B, Liu X. et al. Histone acetyltransferases CBP/p300 in tumorigenesis and CBP/p300 inhibitors as promising novel anticancer agents. *Theranostics* 2022;12:4935–48. 35836809 10.7150/thno.73223 PMC9274749

[22] Balsalobre A, Drouin J. Pioneer factors as master regulators of the epigenome and cell fate. *Nat Rev Mol Cell Biol* 2022;23:449–64. 35264768 10.1038/s41580-022-00464-z

[23] Zaret KS . Pioneer transcription factors initiating gene network changes. *Annu Rev Genet* 2020;54:367–85. 32886547 10.1146/annurev-genet-030220-015007 PMC7900943

[24] Ahmad K, Brahma S, Henikoff S. Epigenetic pioneering by SWI/SNF family remodelers. *Mol Cell* 2024;84:194–201. 38016477 10.1016/j.molcel.2023.10.045 PMC10842064

[25] Vierbuchen T, Ling E, Cowley CJ. et al. AP-1 transcription factors and the BAF complex mediate signal-dependent enhancer selection. *Mol Cell* 2017;68:1067–82. 29272704 10.1016/j.molcel.2017.11.026 PMC5744881

[26] Wolf BK, Zhao YD, McCray A. et al. Cooperation of chromatin remodeling SWI/SNF complex and pioneer factor AP-1 shapes 3D enhancer landscapes. *Nat Struct Mol Biol* 2023;30:10–21. 36522426 10.1038/s41594-022-00880-x PMC10513740

[27] Vernimmen D, Bickmore WA. The hierarchy of transcriptional activation: From enhancer to promoter. *Trends Genet* 2015;31:696–708. 26599498 10.1016/j.tig.2015.10.004

[28] Allen BL, Taatjes DJ. The mediator complex: A central integrator of transcription. *Nat Rev Mol Cell Biol* 2015;16:155–66. 25693131 10.1038/nrm3951 PMC4963239

[29] Kvon EZ, Waymack R, Elabd MG. et al. Enhancer redundancy in development and disease. *Nat Rev Genet* 2021;22:324–36. 33442000 10.1038/s41576-020-00311-x PMC8068586

[30] Mendoza-Figueroa MS, Tatomer DC, Wilusz JE. The integrator complex in transcription and development. *Trends Biochem Sci* 2020;45:923–34. 32800671 10.1016/j.tibs.2020.07.004 PMC7572659

[31] Wan LL, Li WC, Meng Y. et al. Inflammatory immune-associated eRNA: Mechanisms, functions and therapeutic prospects. *Front Immunol* 2022;13:849451. 35514959 10.3389/fimmu.2022.849451 PMC9063412

[32] Kim TK, Hemberg M, Gray JM. Enhancer RNAs: A class of long noncoding RNAs synthesized at enhancers. *Cold Spring Harb Perspect Biol* 2015;7:a018622. 25561718 10.1101/cshperspect.a018622 PMC4292161

[33] Harrison LJ, Bose D. Enhancer RNAs step forward: New insights into enhancer function. *Development* 2022;149:dev200398. 36039999 10.1242/dev.200398 PMC9481971

[34] Akiki RM, Cornbrooks RG, Magami K. et al. A long noncoding eRNA forms R-loops to shape emotional experience-induced behavioral adaptation. *Science* 2024;386:1282–9. 39666799 10.1126/science.adp1562 PMC12071198

[35] Lee JH, Wang RY, Xiong F. et al. Enhancer RNA m6A methylation facilitates transcriptional condensate formation and gene activation. *Mol Cell* 2021;81:3368–85. 34375583 10.1016/j.molcel.2021.07.024 PMC8383322

[36] An YY, Duan H. The role of m6A RNA methylation in cancer metabolism. *Mol Cancer* 2022;21:14. 35022030 10.1186/s12943-022-01500-4 PMC8753874

[37] Pefanis E, Wang JG, Rothschild G. et al. RNA exosome-regulated long non-coding RNA transcription controls super-enhancer activity. *Cell* 2015;161:774–89. 25957685 10.1016/j.cell.2015.04.034 PMC4428671

[38] Wahba L, Gore SK, Koshland D. The homologous recombination machinery modulates the formation of RNA-DNA hybrids and associated chromosome instability. *Elife* 2013;2:e00505. 23795288 10.7554/eLife.00505 PMC3679537

[39] Liang L, Cao CC, Ji L. et al. Complementary Alu sequences mediate enhancer-promoter selectivity. *Nature* 2023;619:868–75. 37438529 10.1038/s41586-023-06323-x

[40] Li WB, Notani D, Ma Q. et al. Functional roles of enhancer RNAs for oestrogen-dependent transcriptional activation. *Nature* 2013;498:516–20. 23728302 10.1038/nature12210 PMC3718886

[41] Rothschild G, Basu U. Lingering questions about enhancer RNA and enhancer transcription-coupled genomic instability. *Trends Genet* 2017;33:143–54. 28087167 10.1016/j.tig.2016.12.002 PMC5291171

[42] Tsai PF, Dell'Orso S, Rodriguez J. et al. A muscle-specific enhancer RNA mediates Cohesin recruitment and regulates transcription In trans. *Mol Cell* 2018;71:129–41. 29979962 10.1016/j.molcel.2018.06.008 PMC6082425

[43] Chen Y, Tan F, Fang Q. et al. Gastrula-Premarked posterior enhancer primes posterior tissue development through cross-talk with TGF-β Signaling pathway. *Advanced Science* 2025;12:e00895. 40583223 10.1002/advs.202500895 PMC12463030

[44] Soutourina J . Transcription regulation by the mediator complex. *Nat Rev Mol Cell Biol* 2018;19:262–74. 29209056 10.1038/nrm.2017.115

[45] Darrow EM, Chadwick BP. Boosting transcription by transcription: Enhancer-associated transcripts. *Chromosome Res* 2013;21:713–24. 24178450 10.1007/s10577-013-9384-6 PMC3867273

[46] Huang ZY, Du GZ, Huang XM. et al. The enhancer RNA lnc-SLC4A1-1 epigenetically regulates unexplained recurrent pregnancy loss (URPL) by activating CXCL8 and NF-kB pathway. *Ebiomedicine* 2018;38:162–70. 30448228 10.1016/j.ebiom.2018.11.015 PMC6306333

[47] Postepska-Igielska A, Giwojna A, Gasri-Plotnitsky L. et al. LncRNA Khps1 regulates expression of the proto-oncogene SPHK1 via triplex-mediated changes in chromatin structure. *Mol Cell* 2015;60:626–36. 26590717 10.1016/j.molcel.2015.10.001

[48] Bose DA, Donahue G, Reinberg D. et al. RNA binding to CBP stimulates histone acetylation and transcription. *Cell* 2017;168:135–149.e122. 28086087 10.1016/j.cell.2016.12.020 PMC5325706

[49] Carullo NVN, Phillips RA, Simon RC. et al. Enhancer RNAs predict enhancer-gene regulatory links and are critical for enhancer function in neuronal systems. *Nucleic Acids Res* 2020;48:9550–70. 32810208 10.1093/nar/gkaa671 PMC7515708

[50] Rahnamoun H, Lee J, Sun ZX. et al. RNAs interact with BRD4 to promote enhanced chromatin engagement and transcription activation. *Nat Struct Mol Biol* 2018;25:687–97. 30076409 10.1038/s41594-018-0102-0 PMC6859054

[51] Narita T, Yamaguchi Y, Yano K. et al. Human transcription elongation factor NELF: Identification of novel subunits and reconstitution of the functionally active complex. *Mol Cell Biol* 2003;23:1863–73. 12612062 10.1128/MCB.23.6.1863-1873.2003 PMC149481

[52] Su BG, Vos SM. Distinct negative elongation factor conformations regulate RNA polymerase II promoter-proximal pausing. *Mol Cell* 2024;84:1243–56. 38401543 10.1016/j.molcel.2024.01.023 PMC10997474

[53] Price DH . P-TEFb, a cyclin-dependent kinase controlling elongation by RNA polymerase II. *Mol Cell Biol* 2000;20:2629–34. 10733565 10.1128/mcb.20.8.2629-2634.2000 PMC85478

[54] Fujinaga K, Huang F, Peterlin BM. P-TEFb: The master regulator of transcription elongation. *Mol Cell* 2023;83:393–403. 36599353 10.1016/j.molcel.2022.12.006 PMC9898187

[55] Rawat P, Boehning M, Hummel B. et al. Stress-induced nuclear condensation of NELF drives transcriptional downregulation. *Mol Cell* 2021;81:1013–26. 33548202 10.1016/j.molcel.2021.01.016 PMC7939545

[56] Gorbovytska V, Kim SK, Kuybu F. et al. Enhancer RNAs stimulate pol II pause release by harnessing multivalent interactions to NELF. *Nat Commun* 2022;13:2429. 35508485 10.1038/s41467-022-29934-w PMC9068813

[57] Zhao Y, Wang LG, Ren SC. et al. Activation of P-TEFb by androgen receptor-regulated enhancer RNAs in castration-resistant prostate cancer. *Cell Rep* 2016;15:599–610. 27068475 10.1016/j.celrep.2016.03.038 PMC5395199

[58] Crossley MP, Song CL, Bocek MJ. et al. R-loop-derived cytoplasmic RNA-DNA hybrids activate an immune response. *Nature* 2023;613:187–94. 36544021 10.1038/s41586-022-05545-9 PMC9949885

[59] Petermann E, Lan L, Zou L. Sources, resolution and physiological relevance of R-loops and RNA-DNA hybrids. *Nat Rev Mol Cell Biol* 2022;23:521–40. 35459910 10.1038/s41580-022-00474-x

[60] Westover KR, Jin P, Yao B. Bridging the gap: R-loop mediated genomic instability and its implications in neurological diseases. *Epigenomics* 2024;16:589–608. 38530068 10.2217/epi-2023-0379 PMC11160457

[61] García-Muse T, Aguilera A. R loops: From physiological to pathological roles. *Cell* 2019;179:604–18. 31607512 10.1016/j.cell.2019.08.055

[62] Chen Q, Zeng Y, Kang J. et al. Enhancer RNAs in transcriptional regulation: Recent insights. *Frontiers in Cell and Developmental Biology* 2023;11:1205540. 37266452 10.3389/fcell.2023.1205540 PMC10229774

[63] Zhou Y, Xu S, Zhang M. et al. Systematic functional characterization of antisense eRNA of protocadherin α composite enhancer. *Genes Dev* 2021;35:1383–94. 34531317 10.1101/gad.348621.121 PMC8494205

[64] Grandi FC, Modi H, Kampman L. et al. Chromatin accessibility profiling by ATAC-seq. *Nat Protoc* 2022;17:1518–52. 35478247 10.1038/s41596-022-00692-9 PMC9189070

[65] Buenrostro JD, Giresi PG, Zaba LC. et al. Transposition of native chromatin for fast and sensitive epigenomic profiling of open chromatin. *DNA-binding proteins and nucleosome position, Nature Methods* 2013;10:1213–8. 24097267 10.1038/nmeth.2688 PMC3959825

[66] Park PJ . ChIP-seq: Advantages and challenges of a maturing technology. *Nat Rev Genet* 2009;10:669–80. 19736561 10.1038/nrg2641 PMC3191340

[67] Johnson DS, Mortazavi A, Myers RM. et al. Genome-wide mapping of in vivo protein-DNA interactions. *Science* 2007;316:1497–502. 17540862 10.1126/science.1141319

[68] Davies JOJ, Oudelaar AM, Higgs DR. et al. How best to identify chromosomal interactions: A comparison of approaches. *Nat Methods* 2017;14:125–34. 28139673 10.1038/nmeth.4146

[69] Gilgenast TG, Phillips-Cremins JE. Systematic evaluation of statistical methods for identifying looping interactions in 5C data. *Cell Systems* 2019;8:197–211. 30904376 10.1016/j.cels.2019.02.006 PMC6696950

[70] Lafontaine DL, Yang LY, Dekker J. et al. Hi-C 3.0: Improved protocol for genome-wide chromosome conformation capture. *Current Protocols* 2021;1:e198. 34286910 10.1002/cpz1.198 PMC8362010

[71] Belton JM, McCord RP, Gibcus JH. et al. Hi-C: A comprehensive technique to capture the conformation of genomes. *Methods* 2012;58:268–76. 22652625 10.1016/j.ymeth.2012.05.001 PMC3874846

[72] Aljogol D, Thompson IR, Osborne CS. et al. Comparison of capture hi-C analytical pipelines. *Front Genet* 2022;13:786501. 35198004 10.3389/fgene.2022.786501 PMC8859814

[73] Liu T, Wang Z. DeepChIA-PET: Accurately predicting ChIA-PET from hi-C and ChIP-seq with deep dilated networks. *PLoS Comput Biol* 2023;19:e1011307. 37440599 10.1371/journal.pcbi.1011307 PMC10368233

[74] Li GL, Ruan XA, Auerbach RK. et al. Extensive promoter-Centered chromatin interactions provide a topological basis for transcription regulation. *Cell* 2012;148:84–98. 22265404 10.1016/j.cell.2011.12.014 PMC3339270

[75] Capurso D, Tang ZH, Ruan YJ. Methods for comparative ChIA-PET and hi-C data analysis. *Methods* 2020;170:69–74. 31629084 10.1016/j.ymeth.2019.09.019

[76] Hayashi T, Ozaki H, Sasagawa Y. et al. Single-cell full-length total RNA sequencing uncovers dynamics of recursive splicing and enhancer RNAs. *Nat Commun* 2018;9:619. 29434199 10.1038/s41467-018-02866-0 PMC5809388

[77] Cardiello JF, Sanchez GJ, Allen MA. et al. Lessons from eRNAs: Understanding transcriptional regulation through the lens of nascent RNAs. *Transcription* 2020;11:3–18. 31856658 10.1080/21541264.2019.1704128 PMC7053884

[78] Magnuson B, Veloso A, Kirkconnell KS. et al. Identifying transcription start sites and active enhancer elements using BruUV-seq. *Sci Rep* 2015;5:17978. 26656874 10.1038/srep17978 PMC4675984

[79] Shibayama Y, Fanucchi S, Mhlanga MM. Visualization of enhancer-derived noncoding RNA. *Methods Mol Biol* 2017;1468:19–32. 27662867 10.1007/978-1-4939-4035-6_3

[80] Ma S, Wang ZX, Xiong ZZ. et al. Enhancer transcription profiling reveals an enhancer RNA-driven ferroptosis and new therapeutic opportunities in prostate cancer. *Signal Transduct Target Ther* 2025;10:87. 40082405 10.1038/s41392-025-02170-6 PMC11906896

[81] Boudreault S, Armero VES, Scott MS. et al. The Epstein-Barr virus EBNA1 protein modulates the alternative splicing of cellular genes. *Virol J* 2019;16:29. 30832682 10.1186/s12985-019-1137-5 PMC6399920

[82] Fulco CP, Munschauer M, Anyoha R. et al. Systematic mapping of functional enhancer-promoter connections with CRISPR interference. *Science* 2016;354:769–73. 27708057 10.1126/science.aag2445 PMC5438575

[83] Mousavi K, Zare H, Dell'orso S. et al. eRNAs promote transcription by establishing chromatin accessibility at defined genomic loci. *Mol Cell* 2013;51:606–17. 23993744 10.1016/j.molcel.2013.07.022 PMC3786356

[84] Zhang Z, Lee JH, Ruan H. et al. Transcriptional landscape and clinical utility of enhancer RNAs for eRNA-targeted therapy in cancer. *Nat Commun* 2019;10:4562. 31594934 10.1038/s41467-019-12543-5 PMC6783481

[85] Zhang Z, Hong W, Ruan H. et al. HeRA: An atlas of enhancer RNAs across human tissues. *Nucleic Acids Res* 2021;49:D932–8. 33119754 10.1093/nar/gkaa940 PMC7779069

[86] Chen H, Liang H. A high-resolution map of human enhancer RNA loci characterizes super-enhancer activities in cancer. *Cancer Cell* 2020;38:701–15. 33007258 10.1016/j.ccell.2020.08.020 PMC7658066

[87] Zhang Z, Luo M, Li Q. et al. Genetic, pharmacogenomic, and immune landscapes of enhancer RNAs across human cancers. *Cancer Res* 2022;82:785–90. 35022213 10.1158/0008-5472.CAN-21-2058

[88] Song C, Zhang GR, Mu XX. et al. eRNAbase: A comprehensive database for decoding the regulatory eRNAs in human and mouse. *Nucleic Acids Res* 2023;52:D81–91. 10.1093/nar/gkad925 PMC10767853 37889077

[89] Yuan J, Tong Y, Liu X. et al. eaQTLdb: An atlas of enhancer activity quantitative trait loci across cancer types. *Int J Cancer* 2023;153:111–9. 36840614 10.1002/ijc.34481

[90] Zhang Y, Gong L, Ding R. et al. eRNA-IDO: A one-stop platform for identification, interactome discovery, and functional annotation of enhancer RNAs. *Genomics Proteomics Bioinformatics* 2024;22:qzae059. 39178387 10.1093/gpbjnl/qzae059 PMC11514848

[91] Cai YM, Lu ZQ, Li B. et al. Genome-wide enhancer RNA profiling adds molecular links between genetic variation and human cancers. *Mil Med Res* 2024;11:36. 38863031 10.1186/s40779-024-00539-2 PMC11165858

[92] Chen J, Wang Z, Hu X. et al. BET inhibition attenuates helicobacter pylori-induced inflammatory response by suppressing inflammatory gene transcription and enhancer activation. *The Journal of Immunology* 2016;196:4132–42. 27084101 10.4049/jimmunol.1502261 PMC4868794

[93] Bojesen RD, Riis LB, Høgdall E. et al. Inflammatory bowel disease and small bowel cancer risk, clinical characteristics, and histopathology: A population-based study. *Clin Gastroenterol Hepatol* 2017;15:1900–7. 28694132 10.1016/j.cgh.2017.06.051

[94] Boyd M, Thodberg M, Vitezic M. et al. Characterization of the enhancer and promoter landscape of inflammatory bowel disease from human colon biopsies. *Nat Commun* 2018;9:1661. 29695774 10.1038/s41467-018-03766-z PMC5916929

[95] Fan Z, Chen X, Liu L. et al. Association of the Polymorphism rs13259960 in SLEAR with predisposition to systemic lupus erythematosus. *Arthritis & Rheumatology* 2020;72:985–96. 31930717 10.1002/art.41200

[96] Fang K, Huang W, Sun YM. et al. Cis-acting lnc-eRNA SEELA directly binds histone H4 to promote histone recognition and leukemia progression. *Genome Biol* 2020;21:269. 33143730 10.1186/s13059-020-02186-x PMC7607629

[97] Wang J, Xiu M, Wang J. et al. METTL16-SENP3-LTF axis confers ferroptosis resistance and facilitates tumorigenesis in hepatocellular carcinoma. *J Hematol Oncol* 2024;17:78. 39218945 10.1186/s13045-024-01599-6 PMC11367782

[98] Wang J, Xiao Q, Cai Y. et al. ABCA1-super enhancer RNA promotes cholesterol efflux, reduces macrophage-mediated inflammation and atherosclerosis, JACC: Basic to translational. *Science* 2024;9:1388–405. 10.1016/j.jacbts.2024.08.005 PMC11733767 39822602

[99] He Y, Cai Y, Cao Y. et al. Application strategies of super-enhancer RNA in cardiovascular diseases. *Biomedicines* 2025;13:117. 39857701 10.3390/biomedicines13010117 PMC11762524

[100] Liao X, Kennel PJ, Liu B. et al. Effect of mechanical unloading on genome-wide DNA methylation profile of the failing human heart. *JCI Insight* 2023;8:e161788. 36656640 10.1172/jci.insight.161788 PMC9977498

[101] Le Gras S, Keime C, Anthony A. et al. Altered enhancer transcription underlies Huntington's disease striatal transcriptional signature. *Sci Rep* 2017;7:42875. 28225006 10.1038/srep42875 PMC5320509

[102] Wan M, Liu Y, Li D. et al. The enhancer RNA, AANCR, regulates APOE expression in astrocytes and microglia. *Nucleic Acids Res* 2024;52:10235–54. 39162226 10.1093/nar/gkae696 PMC11417409

